# Linear energy transfer (LET) distribution outside small radiotherapy field edges produced by 6 MV X-rays

**DOI:** 10.1038/s41598-023-44409-8

**Published:** 2023-12-06

**Authors:** Y. Huerta-Juan, N. Xicohténcatl-Hernández, G. Massillon-JL

**Affiliations:** 1 Instituto de Física, Universidad Nacional Autónoma de México, 04510 Mexico City, Mexico; 2 Departamento de Matemáticas, Universidad Popular Autónoma del Estado de Puebla, 72410 Puebla, Mexico

**Keywords:** Physics, Applied physics, Biological physics

## Abstract

In modern radiotherapy with photons, the absorbed dose outside the radiation field is generally investigated. But it is well known that the biological damage depends not only on the absorbed dose but also on LET. This work investigated the dose-average LET (*L*_*Δ,D*_) outside several small radiotherapy fields to provide information that can help for better evaluating the biological effect in organs at risk close to the tumour volume. The electron fluences produced in liquid water by a 6 MV X-rays Varian iX linac were calculated using the EGSnrc Monte Carlo code. With the electron spectra, *L*_*Δ,D*_ calculations were made for eight open small square fields and the reference field at water depths of 0.15 cm, 1.35 cm, 9.85 cm and 19.85 cm and several off-axis distances. The variation of *L*_*Δ,D*_ from the centre of the beam to 2 cm outside the field’s edge depends on the field size and water depth. Using radiobiological data reported in the literature for chromosomal aberrations as an endpoint for the induction of dicentrics determined in Human Lymphocytes, we estimated the maximum low-dose relative biological effectiveness, (RBE_M_) finding an increase of up to 100% from the centre of the beam to 2 cm from the field's edge.

## Introduction

Track-average (*L*_*Δ,T*_) and dose-average (*L*_*Δ,D*_) linear energy transfer (LET)^1^ are two non-stochastic quantities that have been proposed by the International Commission on Radiation Units and Measurements^2–4^ to describe the quality of an ionizing radiation beam. *L*_*Δ,T*_ is the average energy lost by charged particles due to collisions in crossing a certain distance with energy transfers less than some specified energy cutoff value, Δ, while *L*_*Δ,D*_ corresponds to the average LET associated with the absorbed dose distribution^2^. But for proton therapy, *L*_*Δ,D*_ has been reported to be more suitable for studying the biological effectiveness instead of *L*_*Δ,T*_^5,6^ due to the fact that during the interaction with the cellular or sub-cellular target, the number of proton tracks per cell is considerable at doses therapeutically relevant^5^. Similarly, from a dosimetric point of view, *L*_*Δ,D*_ has been considered as a better parameter to describe the response of different dosimeters^7,8^ since it shows a better relationship between the LET distribution and the dosimeter’s response. In radiotherapy with protons, research has been made to investigate not only the absorbed dose outside of the field^9,10^ but also the *L*_*Δ,D*_ distribution in organs at risk close to the tumour volume^11,12^. In addition, concerns have been expressed related to the clinical consequence of the *L*_*Δ,D*_ level surrounding tumour volumes and principally in organs at risk adjacent to the tumour^11,12^. In modern radiotherapy techniques with photons, there are interests regarding the increase of the survival probability for treated patients to live enough for experimenting with the late radiation effect. For that reason, in contrast to proton therapy, investigations have been performed to determine the absorbed dose levels outside the field size^13–16^ to study any possible radiobiological effect. The radiobiological effect is quantified through the relative biological effectiveness (RBE) which is the ratio of a dose from a reference radiation, (^137^Cs or ^60^Co γ rays) to a dose from a test radiation that gives an identical level of biological effect, and its values vary with the dose, dose fractionation, dose rate, species and biological endpoint considered. Besides the absorbed dose, LET should be considered since it is directly related to biological effectiveness. Concerning LET distribution outside the field size for radiotherapy with photons, Kirkby et al.^17^ have calculated *L*_*Δ,T*_ of the total electron fluence generated by a 6 MV X-ray beam. They reported an important contribution of low energy electrons outside of the primary field whose *L*_*Δ,T*_ values vary from 0.22 to 0.37 keV/μm at 2 cm from the beam’s edge for a 10 × 10 cm^2^ field which are similar to those observed for ^137^Cs photon source^17^. They also conclude that RBE can augment by approximately 25% at 2 cm from the beam’s edge^17^. But, to the best of our knowledge, there is no investigation about *L*_*Δ,D*_ distribution outside small field sizes in radiotherapy with photons.

Recent studies have revealed that *L*_*Δ,D*_ of low-energy secondary electron (produced by electron–electron interactions) spectra generated by photons are a good parameter to describe a dosimeter response in terms of ionization density^7,18,19^. So, for small radiotherapy fields, *L*_*Δ,D*_ of secondary electrons outside of the fields should be taken into account in the evaluation of the possible late effect of secondary radiation on healthy organs close to the tumour volume. This work aimed at investigating the beam characteristics outside of several small radiotherapy fields from 0.7 cm × 0.7 cm up to 4.5 cm × 4.5 cm and the reference 10 cm × 10 cm field in terms of dose-average LET,* L*_*Δ,D*_. We calculated the *L*_*Δ,D*_ for the total electron fluences (TEF: all primary electrons generated by photons + secondary electrons due to electron–electron interactions) and secondary electron (SE: electrons due to electron–electron interactions). Chromosomal aberration is a disorder characterized by a morphological change (deletions, inversions and exchanges) or numerical alteration (gains and losses) in single or multiple chromosomes. Due to their potential to cause stochastic effects, chromosomal aberrations are considered of interest as a biological endpoint. Thus, to put into perspective the LET values obtained in this work, we used chromosomal aberrations results published by Schmid and colleagues^20^ to predict the maximum low-dose relative biological effectiveness (RBE_M_) for the induction of dicentric determined in Human Lymphocytes.

## Materials and methods

The total (TEF) and secondary (SE) electron spectra produced by a 6 MV X-rays Varian iX linac in liquid water were calculated for eight open small square fields of 0.7 × 0.7 cm^2^, 0.9 × 0.9 cm^2^, 1.8 × 1.8 cm^2^, 2.2 × 2.2 cm^2^, 2.7 × 2.7 cm^2^, 3.1 × 3.1 cm^2^, 3.6 × 3.6 cm^2^, 4.5 × 4.5 cm^2^ and the reference field of 10 × 10 cm^2^. The spectra were obtained at 0.15 cm, 1.35 cm, 9.85 cm and 19.85 cm water depths and several off-axis distances (distances outside the central axis) using the FLURZnrc module of the EGSnrc^21^ Monte Carlo code. The calculations were made at 100 cm source-to-surface distance (SSD). Information about the generation of the phase space files and the benchmarking process are reported in our previous work^22^. In the simulations, 5 × 10^10^ histories were followed. The electron transport cut-off (ECUT) and photon transport cut-off (PCUT) were 512 keV and 1 keV, respectively. The maximum fractional energy loss per step (ESTEPE) was 0.01% and the cross-section database generated by the XCOM package was considered. The choice of Monte Carlo simulation settings and their impact on the LET calculations has been evaluated and reported previously^18,19^. Using the electron fluences, the dose-average LET, *L*_*Δ,D*_, have been evaluated as:

1 $${L}_{\Delta ,D}=\frac{{\int }_{\Delta }^{{E}_{max}}{L}_{\Delta }^{2}\left(E\right)\Phi \left(\mathrm{E}\right)\mathrm{dE}+{S}^{2}(\Delta )\Phi (\Delta )\Delta }{{\int }_{\Delta }^{{E}_{max}}{\mathrm{L}}_{\Delta }(\mathrm{E})\Phi \left(\mathrm{E}\right)\mathrm{dE}+S(\Delta )\Phi (\Delta )\Delta }$$

For completeness, the track-average LET, ${L}_{\Delta ,T}$, was also evaluated as:

2 $${L}_{\Delta ,T}=\frac{{\int }_{\Delta }^{{E}_{max}}{L}_{\Delta }\left(E\right)\Phi \left(\mathrm{E}\right)\mathrm{dE}+S(\Delta )\Phi (\Delta )\Delta }{{\int }_{\Delta }^{{E}_{max}}\Phi \left(\mathrm{E}\right)\mathrm{dE}+\Phi (\Delta )\Delta },$$

where $S(E)$, ${L}_{\Delta }\left(E\right),$ $E\mathrm{ and \Phi }(E)$ are the calculated unrestricted and restricted stopping power^23^, the electron energy and the electron energy fluence, respectively. $S(\Delta )\Phi (\Delta )\Delta$ corresponds to a correction for electrons with energies that follow below $\Delta$^24^. The LET values were obtained for Δ = 1 keV.

**Figure 1:**
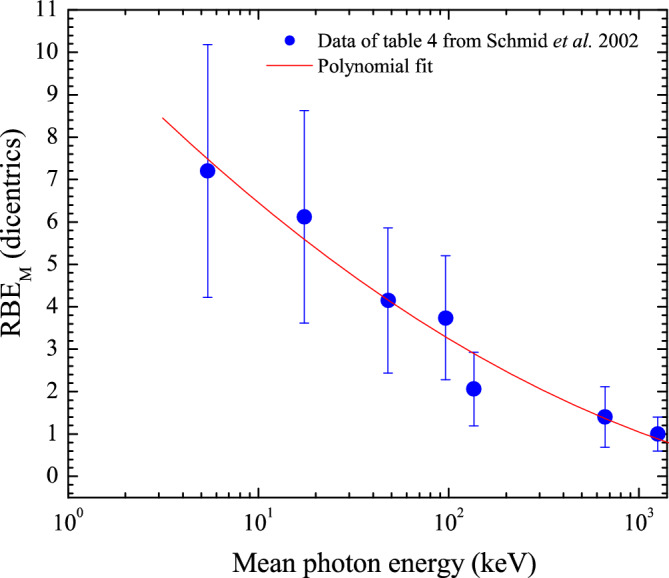
Maximum low-dose RBE_M_ for dicentrics determined in Human Lymphocytes as a function of photon energy reported in Table 4 by Schmid et al.^20^.

As mentioned above, RBE is defined as the ratio of absorbed doses necessary to produce the same biological effect from two radiation beam qualities and depends on the biological system, endpoint, cell type and LET. In this work, the RBE_M_ outside of the fields has been predicted using available radiobiological data reported for chromosomal aberrations as an endpoint for the induction of dicentrics revealed in Human Lymphocytes exposed to a broad range of photon energies^20^. This is done to put into perspective the LET values obtained. For that, the data reported in Table 4 from Schmid et al.^20^. for dicentrics determined in Human Lymphocytes has been fitted. Figure 1 displays the data and a polynomial fit of degree two that describes the data.

Using the polynomial fit, the RBE_M_ was estimated for four water depths and several off-axis distances.

## Results and discussion

**Figure 2:**
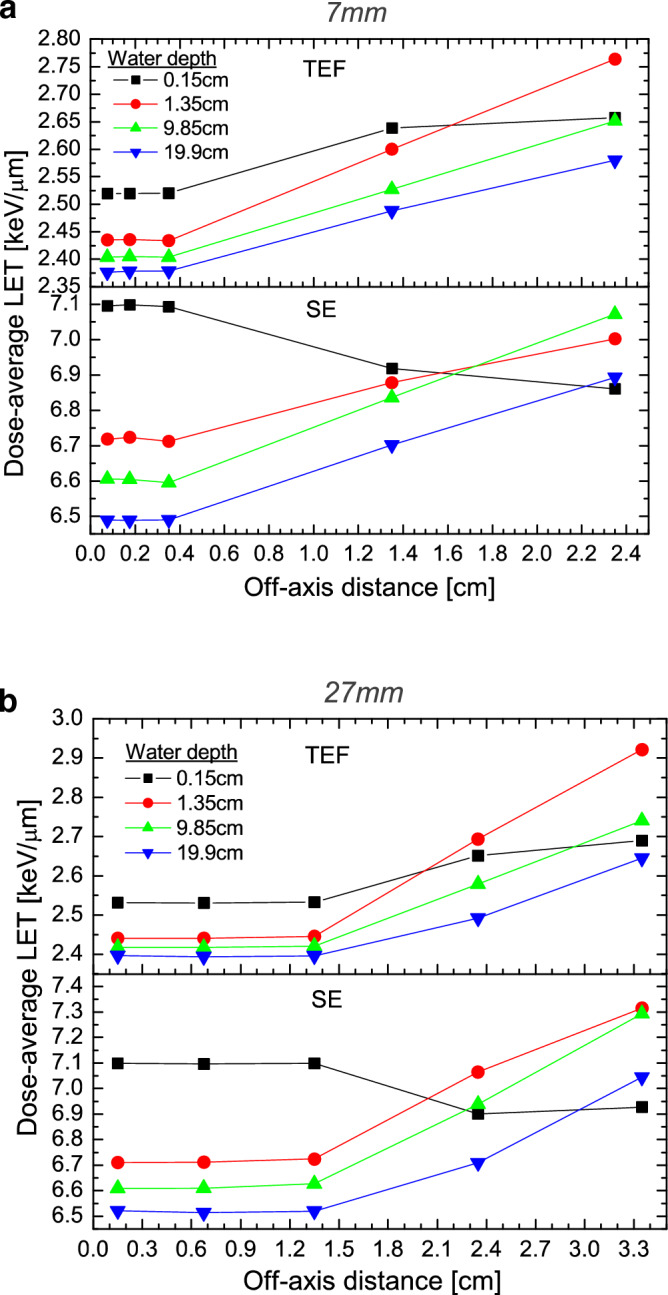
(**a**) Dose-average LET for the field size of 0.7 × 0.7 cm^2^ as a function of the off-axis distance. (**b**) Dose-average LET for the field size of 2.7 × 2.7 cm^2^ as a function of the off-axis distance.

**Figure 3:**
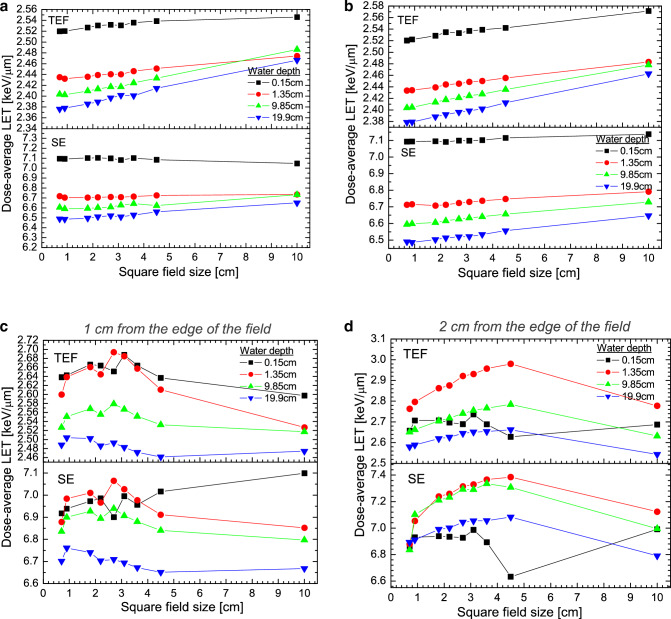
(**a**) Dose-average LET at the centre of the beam as a function of the field size. (**b**) Dose-average LET at the edge of the beam as a function of the field size. (**c**) Dose-average LET at 1 cm from the edge of the beam as a function of the field size. (**d**) Dose-average LET at 2 cm from the edge of the beam as a function of the field size.

Figure 2a and b display the *L*_*Δ,D*_ values as a function of the off-axis distance for the field size of 0.7 × 0.7 cm^2^ and 2.7 × 2.7 cm^2^, respectively. As can be seen, independent of the field size and the electron fluence, *L*_*Δ,D*_ is almost constant within the centre of the beam and increases as the off-axis distance increases. This can be associated with the existence of low photon energy outside of the field’s edge which generated low-energy secondary electrons^22^. Note that in both Fig. 2a and b, at 0.15 cm water depth close to the phantom surface, *L*_*Δ,D*_ for the TEF slightly increases beyond the field’s edge while for the SE, *L*_*Δ,D*_ decreases instead. Such a feature is observed for all the field sizes studied. This is presumably associated with the contribution of high energy electrons coming from the head of the linac which reach the water surface^22^. For the TEF, the variation of *L*_*Δ,D*_ from the centre of the beam to 2 cm outside of the field edge depends on the field size and increases by up to 14–21% at 1.35 cm water depth (close to the maximum dose, d_max_), 10–14% at 9.85 cm water depth, 8.5–11% at 19.85 cm water depth, being greater for larger field size. For the SE spectra, the *L*_*Δ,D*_ varies from 3.4 to 9.7% at 1.35 cm water depth, 3.5% to 10.2% at 9.85 water depth and 6.2% to 8.2% at 19.85 water depth from the centre of the beam to 2 cm outside the field’s edge. Figure 3a–d display the ${L}_{\Delta ,D}$ values for TEF and SE spectra as a function of field size at several depths and off-axis distances. Tables 1, 2, 3 and 4 present the ${L}_{\Delta ,D}$ data for 0.15 cm, 1.35 cm, 9.85 cm and 19.85 cm water depths, respectively. As can be seen in Fig. 3a to b for the centre and the edge of the fields, the shape of the curves of ${L}_{\Delta ,D}$ versus field size are very similar. Whereas, for 1 cm and 2 cm beyond the edge of the field size shown in Fig. 3c and d, ${L}_{\Delta ,D}$ increases, reaches a maximum and thereafter decreases as the field size increases. Note that in Fig. 3a and c, the ${L}_{\Delta ,D}$ decreases as the depth increases. This is expected due to the hardness of the X-ray beams caused by the photon attenuation as the depth augments. But in Fig. 3d, the ${L}_{\Delta ,D}$ at the surface of the beam is smaller than at the other depths. This could be associated with the contribution of high-energy electrons scattered close to the surface of the phantom.

**Table 1:** Track and dose average LET of total fluence (TEF) and secondary electrons (SE) at water depth = 0.15 cm.

Field size (cm × cm)	Off-axis distance (cm)	Track-average LET (keV/µm)		Dose-average LET (keV/µm)		RBE_M_ relative to ^60^Co gamma rays
		TEF	SE	TEF	SE	
0.7 × 0.7	0	0.275	1.886	2.520	7.095	1.08 ± 0.32
0.9 × 0.9	0	0.275	1.885	2.520	7.094	1.08 ± 0.32
1.8 × 1.8	0	0.276	1.888	2.527	7.101	1.09 ± 0.33
2.2 × 2.2	0	0.277	1.885	2.530	7.103	1.10 ± 0.33
2.7 × 2.7	0	0.277	1.879	2.532	7.099	1.10 ± 0.33
3.1 × 3.1	0	0.275	1.843	2.531	7.082	1.10 ± 0.33
3.6 × 3.6	0	0.277	1.880	2.536	7.103	1.11 ± 0.33
4.5 × 4.5	0	0.277	1.850	2.539	7.085	1.12 ± 0.34
10 × 10	0	0.274	1.779	2.546	7.048	1.16 ± 0.34
0.7 × 0.7	0.175	0.275	1.891	2.519	7.098	1.08 ± 0.34
0.9 × 0.9	0.225	0.275	1.894	2.521	7.102	1.08 ± 0.33
1.8 × 1.8	0.45	0.276	1.886	2.526	7.100	1.10 ± 0.33
2.2 × 2.2	0.55	0.277	1.884	2.529	7.101	1.10 ± 0.33
2.7 × 2.7	0.675	0.277	1.875	2.531	7.097	1.10 ± 0.33
3.1 × 3.1	0.775	0.277	1.873	2.531	7.099	1.11 ± 0.33
3.6 × 3.6	0.9	0.277	1.871	2.534	7.096	1.11 ± 0.33
4.5 × 4.5	1.125	0.278	1.877	2.540	7.099	1.12 ± 0.34
10 × 10	2.5	0.282	1.856	2.563	7.109	1.17 ± 0.34
0.7 × 0.7	0.35	0.274	1.882	2.520	7.093	1.08 ± 0.36
0.9 × 0.9	0.45	0.275	1.883	2.522	7.093	1.08 ± 0.32
1.8 × 1.8	0.9	0.276	1.876	2.528	7.095	1.10 ± 0.33
2.2 × 2.2	1.1	0.277	1.864	2.535	7.091	1.12 ± 0.33
2.7 × 2.7	1.35	0.277	1.875	2.533	7.099	1.11 ± 0.33
3.1 × 3.1	1.55	0.278	1.871	2.537	7.098	1.12 ± 0.33
3.6 × 3.6	1.8	0.278	1.876	2.539	7.102	1.12 ± 0.34
4.5 × 4.5	2.25	0.279	1.885	2.542	7.115	1.13 ± 0.34
10 × 10	5.0	0.285	1.883	2.571	7.137	1.19 ± 0.34
0.7 × 0.7	1.35	0.281	1.531	2.638	6.918	1.52 ± 0.46
0.9 × 0.9	1.45	0.281	1.546	2.642	6.939	1.52 ± 0.46
1.8 × 1.8	1.9	0.287	1.571	2.666	6.972	1.70 ± 0.51
2.2 × 2.2	2.1	0.288	1.591	2.664	6.985	1.69 ± 0.51
2.7 × 2.7	2.35	0.279	1.504	2.651	6.900	1.79 ± 0.54
3.1 × 3.1	2.55	0.290	1.583	2.688	6.995	1.80 ± 0.54
3.6 × 3.6	2.8	0.286	1.568	2.664	6.956	1.71 ± 0.51
4.5 × 4.5	3.25	0.283	1.669	2.637	7.016	1.48 ± 0.44
10 × 10	6.0	0.285	1.799	2.597	7.099	1.28 ± 0.44
0.7 × 0.7	2.35	0.275	1.491	2.657	6.860	1.58 ± 0.50
0.9 × 0.9	2.45	0.289	1.502	2.707	6.930	1.64 ± 0.49
1.8 × 1.8	2.9	0.289	1.516	2.708	6.939	1.82 ± 0.55
2.2 × 2.2	3.1	0.286	1.521	2.697	6.935	1.85 ± 0.55
2.7 × 2.7	3.35	0.284	1.516	2.689	6.927	1.93 ± 0.58
3.1 × 3.1	3.55	0.296	1.549	2.737	6.986	1.95 ± 0.58
3.6 × 3.6	3.8	0.282	1.487	2.689	6.893	1.99 ± 0.60
4.5 × 4.5	4.25	0.261	1.255	2.628	6.632	2.05 ± 0.61
10 × 10	7.0	0.287	1.596	2.687	6.990	1.73 ± 0.61

We also include the predicted RBE_M_ for dicentrics determined in human lymphocytes. The combined standard uncertainty of 0.6% (coverage factor *k* = *1*)^25^.

**Table 2:** Track and dose average LET of total fluence (TEF) and secondary electrons (SE) at water depth = 1.35 cm.

Field size (cm × cm)	Off-axis distance (cm)	Track-average LET (keV/µm)		Dose-average LET (keV/µm)		RBE_M_ relative to ^60^Co gamma rays
		TEF	SE	TEF	SE	
0.7 × 0.7	0	0.242	1.476	2.435	6.718	1.07 ± 0.32
0.9 × 0.9	0	0.241	1.462	2.432	6.704	1.08 ± 0.32
1.8 × 1.8	0	0.242	1.461	2.436	6.704	1.10 ± 0.33
2.2 × 2.2	0	0.242	1.461	2.439	6.706	1.10 ± 0.33
2.7 × 2.7	0	0.243	1.463	2.441	6.710	1.11 ± 0.33
3.1 × 3.1	0	0.242	1.462	2.440	6.708	1.11 ± 0.33
3.6 × 3.6	0	0.244	1.465	2.446	6.714	1.13 ± 0.34
4.5 × 4.5	0	0.245	1.455	2.451	6.725	1.13 ± 0.34
10 × 10	0	0.248	1.478	2.474	6.735	1.13 ± 0.34
0.7 × 0.7	0.175	0.242	1.477	2.436	6.724	1.07 ± 0.34
0.9 × 0.9	0.225	0.242	1.473	2.435	6.716	1.08 ± 0.32
1.8 × 1.8	0.45	0.242	1.462	2.436	6.704	1.10 ± 0.33
2.2 × 2.2	0.55	0.242	1.463	2.438	6.707	1.10 ± 0.33
2.7 × 2.7	0.675	0.243	1.467	2.441	6.711	1.11 ± 0.33
3.1 × 3.1	0.775	0.243	1.462	2.443	6.708	1.11 ± 0.33
3.6 × 3.6	0.9	0.244	1.477	2.446	6.724	1.12 ± 0.34
4.5 × 4.5	1.125	0.245	1.472	2.449	6.723	1.14 ± 0.34
10 × 10	2.5	0.250	1.504	2.478	6.771	1.14 ± 0.34
0.7 × 0.7	0.35	0.241	1.472	2.434	6.712	1.08 ± 0.32
0.9 × 0.9	0.45	0.241	1.473	2.434	6.716	1.08 ± 0.32
1.8 × 1.8	0.9	0.242	1.464	2.439	6.708	1.11 ± 0.33
2.2 × 2.2	1.1	0.243	1.466	2.444	6.713	1.12 ± 0.34
2.7 × 2.7	1.35	0.244	1.475	2.446	6.724	1.12 ± 0.34
3.1 × 3.1	1.55	0.245	1.479	2.448	6.730	1.12 ± 0.34
3.6 × 3.6	1.8	0.245	1.484	2.450	6.737	1.13 ± 0.34
4.5 × 4.5	2.25	0.246	1.493	2.455	6.747	1.14 ± 0.34
10 × 10	5.0	0.253	1.517	2.483	6.792	1.14 ± 0.34
0.7 × 0.7	1.35	0.278	1.526	2.600	6.878	1.63 ± 0.49
0.9 × 0.9	1.45	0.292	1.611	2.639	6.984	1.62 ± 0.49
1.8 × 1.8	1.9	0.296	1.630	2.661	7.010	1.79 ± 0.54
2.2 × 2.2	2.1	0.290	1.592	2.644	6.966	1.76 ± 0.53
2.7 × 2.7	2.35	0.304	1.664	2.694	7.065	1.86 ± 0.56
3.1 × 3.1	2.55	0.301	1.627	2.685	7.026	1.85 ± 0.56
3.6 × 3.6	2.8	0.293	1.591	2.657	6.976	1.75 ± 0.52
4.5 × 4.5	3.25	0.281	1.560	2.611	6.911	1.52 ± 0.46
10 × 10	6.0	0.261	1.549	2.527	6.852	1.52 ± 0.46
0.7 × 0.7	2.35	0.311	1.526	2.764	6.878	1.74 ± 0.56
0.9 × 0.9	2.45	0.321	1.539	2.796	7.054	1.77 ± 0.53
1.8 × 1.8	2.9	0.350	1.703	2.863	7.239	1.92 ± 0.58
2.2 × 2.2	3.1	0.355	1.719	2.876	7.259	1.95 ± 0.58
2.7 × 2.7	3.35	0.371	1.752	2.922	7.315	2.02 ± 0.61
3.1 × 3.1	3.55	0.374	1.756	2.931	7.330	2.04 ± 0.61
3.6 × 3.6	3.8	0.385	1.773	2.957	7.366	2.08 ± 0.62
4.5 × 4.5	4.25	0.393	1.762	2.980	7.386	2.11 ± 0.63
10 × 10	7.0	0.320	1.673	2.778	7.124	2.11 ± 0.63

We also include the predicted RBE_M_ for dicentrics determined in human lymphocytes. The combined standard uncertainty of 0.6% (coverage factor *k* = *1*)^25^.

**Table 3:** Track and dose average LET of total fluence (TEF) and secondary electrons (SE)at water depth = 9.85 cm.

Field size (cm × cm)	Off-axis distance (cm)	Track-average LET (keV/µm)		Dose-average LET (keV/µm)		RBE_M_ relative to ^60^Co gamma rays
		TEF	SE	TEF	SE	
0.7 × 0.7	0	0.231	1.389	2.404	6.605	1.01 ± 0.30
0.9 × 0.9	0	0.231	1.374	2.402	6.590	1.01 ± 0.30
1.8 × 1.8	0	0.232	1.381	2.410	6.595	1.03 ± 0.31
2.2 × 2.2	0	0.233	1.382	2.413	6.603	1.04 ± 0.31
2.7 × 2.7	0	0.234	1.388	2.417	6.609	1.06 ± 0.32
3.1 × 3.1	0	0.234	1.400	2.417	6.627	1.06 ± 0.32
3.6 × 3.6	0	0.236	1.412	2.424	6.642	1.08 ± 0.32
4.5 × 4.5	0	0.238	1.379	2.433	6.622	1.10 ± 0.33
10 × 10	0	0.249	1.464	2.486	6.732	1.10 ± 0.33
0.7 × 0.7	0.175	0.232	1.389	2.405	6.605	1.01 ± 0.32
0.9 × 0.9	0.225	0.231	1.382	2.404	6.598	1.01 ± 0.30
1.8 × 1.8	0.45	0.232	1.380	2.409	6.595	1.03 ± 0.31
2.2 × 2.2	0.55	0.233	1.386	2.412	6.604	1.04 ± 0.31
2.7 × 2.7	0.675	0.234	1.390	2.417	6.610	1.06 ± 0.32
3.1 × 3.1	0.775	0.235	1.390	2.420	6.610	1.06 ± 0.32
3.6 × 3.6	0.9	0.236	1.400	2.425	6.626	1.08 ± 0.32
4.5 × 4.5	1.125	0.238	1.417	2.433	6.653	1.10 ± 0.33
10 × 10	2.5	0.249	1.473	2.485	6.741	1.10 ± 0.33
0.7 × 0.7	0.35	0.231	1.379	2.404	6.595	1.01 ± 0.30
0.9 × 0.9	0.45	0.232	1.381	2.404	6.598	1.01 ± 0.30
1.8 × 1.8	0.9	0.234	1.387	2.413	6.606	1.05 ± 0.31
2.2 × 2.2	1.1	0.235	1.394	2.418	6.616	1.06 ± 0.32
2.7 × 2.7	1.35	0.235	1.401	2.421	6.627	1.06 ± 0.32
3.1 × 3.1	1.55	0.236	1.403	2.424	6.634	1.07 ± 0.32
3.6 × 3.6	1.8	0.237	1.410	2.428	6.641	1.08 ± 0.32
4.5 × 4.5	2.25	0.239	1.421	2.435	6.656	1.10 ± 0.33
10 × 10	5.0	0.248	1.465	2.478	6.729	1.10 ± 0.33
0.7 × 0.7	1.35	0.264	1.566	2.527	6.702	1.41 ± 0.42
0.9 × 0.9	1.45	0.272	1.617	2.551	6.761	1.42 ± 0.43
1.8 × 1.8	1.9	0.275	1.641	2.568	6.741	1.52 ± 0.46
2.2 × 2.2	2.1	0.271	1.605	2.556	6.703	1.48 ± 0.44
2.7 × 2.7	2.35	0.278	1.644	2.579	6.710	1.55 ± 0.46
3.1 × 3.1	2.55	0.274	1.612	2.567	6.693	1.49 ± 0.45
3.6 × 3.6	2.8	0.269	1.586	2.551	6.672	1.41 ± 0.42
4.5 × 4.5	3.25	0.263	1.559	2.533	6.651	1.34 ± 0.40
10 × 10	6.0	0.256	1.506	2.517	6.668	1.34 ± 0.40
0.7 × 0.7	2.35	0.298	1.566	2.651	6.836	1.58 ± 0.50
0.9 × 0.9	2.45	0.304	1.763	2.663	7.102	1.61 ± 0.48
1.8 × 1.8	2.9	0.319	1.883	2.705	7.211	1.68 ± 0.51
2.2 × 2.2	3.1	0.323	1.905	2.718	7.232	1.71 ± 0.51
2.7 × 2.7	3.35	0.331	1.967	2.740	7.293	1.76 ± 0.53
3.1 × 3.1	3.55	0.334	1.950	2.755	7.291	1.77 ± 0.53
3.6 × 3.6	3.8	0.340	2.012	2.767	7.336	1.79 ± 0.54
4.5 × 4.5	4.25	0.342	1.960	2.784	7.308	1.83 ± 0.55
10 × 10	7.0	0.284	1.659	2.632	6.995	1.83 ± 0.55

The combined standard uncertainty of 0.6% (coverage factor *k* = *1*)^25^. We also include the predicted RBE_M_ for dicentrics determined in human lymphocytes.

**Table 4:** Track and dose average LET of total fluence (TEF) and secondary electrons (SE) at water depth = 19.9 cm.

Field size (cm × cm)	Off-axis distance (cm)	Track-average LET (keV/µm)		Dose-average LET (keV/µm)		RBE_M_ relative to ^60^Co gamma rays
		TEF	SE	TEF	SE	
0.7 × 0.7	0	0.223	1.307	2.376	6.489	1.00 ± 0.28
0.9 × 0.9	0	0.223	1.300	2.378	6.486	1.00 ± 0.29
1.8 × 1.8	0	0.224	1.307	2.386	6.497	1.00 ± 0.29
2.2 × 2.2	0	0.225	1.320	2.389	6.511	1.00 ± 0.29
2.7 × 2.7	0	0.227	1.327	2.397	6.521	1.00 ± 0.30
3.1 × 3.1	0	0.227	1.308	2.402	6.509	1.01 ± 0.30
3.6 × 3.6	0	0.228	1.330	2.401	6.527	1.02 ± 0.31
4.5 × 4.5	0	0.231	1.334	2.414	6.560	1.05 ± 0.31
10 × 10	0	0.242	1.404	2.466	6.650	1.05 ± 0.31
0.7 × 0.7	0.175	0.223	1.301	2.378	6.488	1.00 ± 0.30
0.9 × 0.9	0.225	0.223	1.301	2.379	6.487	1.00 ± 0.29
1.8 × 1.8	0.45	0.225	1.309	2.385	6.498	1.00 ± 0.29
2.2 × 2.2	0.55	0.225	1.313	2.389	6.504	1.00 ± 0.30
2.7 × 2.7	0.675	0.227	1.323	2.394	6.513	1.00 ± 0.30
3.1 × 3.1	0.775	0.227	1.325	2.397	6.519	1.01 ± 0.30
3.6 × 3.6	0.9	0.228	1.335	2.402	6.535	1.02 ± 0.31
4.5 × 4.5	1.125	0.231	1.351	2.412	6.554	1.05 ± 0.31
10 × 10	2.5	0.242	1.398	2.470	6.650	1.05 ± 0.31
0.7 × 0.7	0.35	0.223	1.304	2.379	6.490	1.00 ± 0.29
0.9 × 0.9	0.45	0.223	1.301	2.379	6.487	1.00 ± 0.29
1.8 × 1.8	0.9	0.225	1.312	2.388	6.504	1.00 ± 0.29
2.2 × 2.2	1.1	0.226	1.319	2.392	6.514	1.00 ± 0.30
2.7 × 2.7	1.35	0.227	1.319	2.396	6.519	1.00 ± 0.30
3.1 × 3.1	1.55	0.228	1.318	2.399	6.521	1.01 ± 0.30
3.6 × 3.6	1.8	0.229	1.331	2.402	6.535	1.02 ± 0.31
4.5 × 4.5	2.25	0.231	1.349	2.412	6.557	1.04 ± 0.31
10 × 10	5.0	0.241	1.401	2.463	6.647	1.04 ± 0.31
0.7 × 0.7	1.35	0.251	1.468	2.448	6.702	1.28 ± 0.38
0.9 × 0.9	1.45	0.256	1.503	2.504	6.761	1.31 ± 0.39
1.8 × 1.8	1.9	0.254	1.485	2.502	6.741	1.34 ± 0.40
2.2 × 2.2	2.1	0.249	1.453	2.486	6.703	1.28 ± 0.38
2.7 × 2.7	2.35	0.251	1.462	2.493	6.710	1.29 ± 0.39
3.1 × 3.1	2.55	0.248	1.444	2.482	6.693	1.24 ± 0.37
3.6 × 3.6	2.8	0.245	1.429	2.471	6.672	1.20 ± 0.36
4.5 × 4.5	3.25	0.242	1.411	2.461	6.651	1.17 ± 0.36
10 × 10	6.0	0.243	1.416	2.474	6.668	1.17 ± 0.35
0.7 × 0.7	2.35	0.275	1.591	2.580	6.893	1.45 ± 0.46
0.9 × 0.9	2.45	0.277	1.608	2.589	6.911	1.46 ± 0.44
1.8 × 1.8	2.9	0.287	1.685	2.620	6.991	1.53 ± 0.46
2.2 × 2.2	3.1	0.289	1.695	2.626	7.002	1.55 ± 0.47
2.7 × 2.7	3.35	0.293	1.739	2.645	7.044	1.58 ± 0.47
3.1 × 3.1	3.55	0.295	1.735	2.651	7.054	1.60 ± 0.48
3.6 × 3.6	3.8	0.297	1.740	2.653	7.056	1.61 ± 0.48
4.5 × 4.5	4.25	0.298	1.757	2.663	7.084	1.64 ± 0.49
10 × 10	7.0	0.259	1.497	2.543	6.791	1.64 ± 0.49

The combined standard uncertainty of 0.6% (coverage factor *k* = *1*)^25^. We also include the predicted RBE_M_ for dicentrics determined in human lymphocytes.

The ${L}_{\Delta ,D}$ values reported in this work for the TEF vary from 2.45 to 2.98 keV/μm at 2 cm from the field’s edge, while for the SE, the values of ${L}_{\Delta ,D}$ are the order of 6.70 keV/μm to 7.40 keV/μm. Such results suggested the importance of considering the secondary electrons generated by photons since they are the main ones responsible for the biological damage of ionizing radiation into the matter.

Also included in Tables 1, 2, 3 and 4 are the ${L}_{\Delta ,T}$ data for 0.15 cm, 1.35 cm, 9.85 cm and 19.85 cm water depths, respectively. The shape of the curves ${L}_{\Delta ,T}$ versus field size (not shown) are comparable to that shown in Fig. 3a–d for ${L}_{\Delta ,D}$ versus field size. That is, ${L}_{\Delta ,T}$ increases as a function of the field size and decreases as the depth decreases. Similar to ${L}_{\Delta ,D}$, ${L}_{\Delta ,T}$ diminishes within the centre of the beam and growths outside of the field’s edge, independent of the electron spectra. But in contrast to ${L}_{\Delta ,D}$, ${L}_{\Delta ,T}$ varies from 0.24 to 0.38 keV/μm at 2 cm from the field’s edge which represents an augmentation of ~ 60%. This would suggest higher biological effects at distances beyond the field’s edge. For the 10 × 10 cm field, the ${L}_{\Delta ,T}$ values obtained in this work are analogous to those reported by Kirkby and colleagues^17^. In this work, the ${L}_{\Delta ,T}$ values at 9.85 cm water depth vary from 0.25 to 0.28 keV/μm at 2 cm from the field’s edge, versus 0.22 keV/μm to 0.37 keV/μm at 5 cm depth reported by Kirkby and colleagues.

The estimated RBE_M_ for dicentrics determined in Human Lymphocytes as a function of off-axis-distance at 0.15 cm, 1.35 cm, 9.85 cm and 19.9 cm water depths are displayed in Tables 1, 2, 3 and 4, respectively. Note the high degree of uncertainty in the RBE_M_ values obtained, which is associated with the large uncertainty in the reference data. Similar to the LET values, within the field size, the RBE_M_ is almost constant and increases as the distance outside of the field’s edge increases. For example, the biological effectiveness at 2 cm outside the field edge can have values up to 2 compared to 1.08 witing the field size (see data for a water depth of 1.35 cm in Table 2). Which represents an increase by a factor of two. Also, observe that RBE_M_ values are larger at 1.35 cm depth than the other depths. This can be explained by the fact that at this depth the beam doesn’t reach the charged particle equilibrium yet. The change in RBE_M_ from the centre of the beam to 2 cm outside the field’s edge depends on the field size and increases by up to 100% at 1.35 cm depth, 80% at 9.85 cm depth, and 60% at 19.85 cm depth. As seen in Tables 1, 2, 3 and 4, the change in RBE from the primary field to 2 cm from the field’s edge is remarkably larger than the variation in LET. This is presumably associated with the limitation of 1 keV as electron transport energy cut-off in the Monte Carlo calculation of the electron fluences. That is, it is possible to follow electrons down to 1 keV. This means one can only follow electrons until the kinetic energy falls to 1 keV and assume that, at energy below, all the energies are deposited locally. Such a limitation can be overcome by introducing in the Monte Carlo code new cross-section data recently reported for very low energy electrons with acceptable accuracy^26^.

## Conclusions

We have investigated the dose-average linear energy transfer (${L}_{\Delta ,D}$) distribution outside the field’s edge of several small radiotherapy beams. In addition, we estimated the maximum low-dose relative biological effectiveness (RBE_M_) for dicentrics determined in Human Lymphocytes using biological data published in the literature. We observed that both RBE_M_ and ${L}_{\Delta ,D}$ are almost constant within the centre of the beam and increase outside the field's edge. From the centre of the primary radiation field to 2 cm from the field's edge, ${L}_{\Delta ,D}$ has a maximum increase of up to 21% while RBE_M_ varies by up to 100%. The RBE data presented here are from in vitro cellular studies which offer the possibility to investigate basic biological responses to radiation. But in clinical radiotherapy, the environment of the cell’s cloud such as organs should be considered, thus more studies have to be done to evaluate how RBE effects observed in vitro can be translated into effects within complete biological systems. One can conclude that the result of this work can be used as a starting point for the elucidation of the clinical implications of LET and RBE in radiotherapy treatment with photons as done for protons^27^.

## Data Availability

The datasets used and/or analysed during the current study are available from the corresponding author on reasonable request.
